# Rational design of heterodimeric receptors capable of activating target signaling molecules

**DOI:** 10.1038/s41598-021-96396-3

**Published:** 2021-08-19

**Authors:** Tatphon Kongkrongtong, Ruolan Zhang, Masahiro Kawahara

**Affiliations:** 1 Department of Chemistry and Biotechnology, Graduate School of Engineering, The University of Tokyo, 7-3-1 Hongo, Bunkyo-ku, Tokyo, 113-8656 Japan; 2 Department of Bioengineering, Graduate School of Engineering, The University of Tokyo, 7-3-1 Hongo, Bunkyo-ku, Tokyo, 113-8656 Japan; 3 Laboratory of Cell Vaccine, Center for Vaccine and Adjuvant Research (CVAR), National Institutes of Biomedical Innovation, Health and Nutrition (NIBIOHN), 7-6-8 Saito-Asagi, Ibaraki-shi, Osaka, 567-0085 Japan

**Keywords:** Biotechnology, Molecular engineering, Protein design, Synthetic biology

## Abstract

Intracellular signal transduction is regulated by a variety of transmembrane receptors. Many researchers have aimed to arbitrarily regulate the intracellular signaling and subsequent cell fate with artificial receptors, of which the ligand recognition and signaling properties could be artificially designed. Although several architectures of homodimeric artificial receptors have been reported, engineering of heterodimeric receptors, which are abundant among natural receptors, have yet to be thoroughly investigated. In this study, we rationally design artificial heterodimeric receptors for activating target signaling molecules. We locate a tyrosine motif on an engineered tyrosine kinase domain, which is further connected to a small molecule-responsive heterodimeric module, attaining a pair of heterodimeric receptors with different tyrosine motifs within the pair. The resultant heterodimeric receptors successfully activate target signaling molecules and even control cell proliferation levels according to the properties of tyrosine motifs connected. Thus, our heterodimeric receptors may open a new era of tailor-made designer receptors, which could be useful for cell therapy against intractable diseases.

## Introduction

Transmembrane receptors play important roles in signal transduction of mammalian cells. The receptors receive signal molecules outside of the cells and subsequently transmit the signals to intracellular signal transduction pathways^1–6^. In particular, type I cytokine receptors and receptor tyrosine kinases, which are single-transmembrane receptors important for controlling basic cell fates such as proliferation and differentiation^7–15^, are activated by recognizing their specific ligands in the extracellular domain, and then tyrosine residues within the intracellular domain of the receptors are phosphorylated, which serves as a scaffold for recruiting specific signaling molecules. While many receptors of these two receptor families are activated by homodimerization, there exist heterodimeric receptors in the type I cytokine receptors such as interleukin-2 receptor (IL-2R), IL-3R, and IL-6R subfamilies, which play key roles in controlling cell fates such as proliferation and differentiation. In these receptor subfamilies, subunits are commonly shared among the receptor members^11^ (*e.g.* γ_c_, β_c_, and gp130 for the IL-2R, IL-3R, IL-6R subfamilies, respectively), which would attain similar signaling properties fine-tuned by different ligands.

Therefore, if the ligand recognition and signaling properties of the type I cytokine receptors and receptor tyrosine kinases could be artificially designed, cell fates including proliferation, differentiation, and migration would be arbitrarily and sophisticatedly controlled by an artificial ligand^16^. This approach would attract much attention in the field of synthetic biology, where elucidating signal transduction and creating desirable functional receptors are important aspects, and also in the field of cell therapy, where efficient growth maintenance and specific lineage differentiation are important for clinical applications^17,18^. With this in mind, we previously implemented two approaches that mimicked homodimeric receptors using modular chimeric receptors with artificial ligand-recognition domains^19–23^. In one approach, we used single-chain Fv as a ligand-recognition domain to trigger signaling via an oligomeric antigen, and the Janus kinase (JAK)-binding domain of a type I cytokine receptor c-mpl as a component for recruiting the tyrosine kinase JAK to the chimeric receptor^19–22^. The chimeric receptor also included tyrosine motifs of interest that can preferentially recruit specific signaling molecules upon tyrosine phosphorylation by the recruited JAK. However, JAK2, which constitutively associates with the JAK-binding domain of c-mpl, can directly recruit STAT5, causing a problem that STAT5 was activated independent of the tyrosine motif connected in the chimeric receptor^20,22^. In another approach, recently we have developed a designer receptor by elaborately engineering a receptor tyrosine kinase c-KIT, which has its own tyrosine kinase domain within the intracellular domain. We used a mutant of FK506-binding protein 12 as a ligand-recognition domain to trigger signaling via a synthetic chemical dimerizer, and connected with an engineered scaffold-less domain derived from the c-KIT intracellular domain and tyrosine motifs of interest^23^. The resultant designer receptor activated only on-target signaling molecules dependent on the tyrosine motifs connected, indicating successful establishment of an artificial receptor platform that could significantly mimic homodimeric receptors.

In contrast to homodimeric receptors, heterodimeric receptors that play important roles as type I cytokine receptors remain to be intensively studied for creating artificial receptors. In this study, we aim to develop a platform for artificially mimicking and designing heterodimeric receptors. We choose the engineered c-KIT^23^, which has been developed as the second approach, to create a heterodimeric designer receptor, because it would be easier to engineer two heterodimeric receptor chains from the already validated engineered c-KIT^23^, rather than to engineer two heterodimeric receptor chains from type I cytokine receptors with completely different lengths and architectures. While tyrosine motifs can be inserted at four positions in the homodimeric engineered c-KIT, the number of tyrosine motifs could be doubled in principle in the heterodimeric engineered c-KIT, which would be advantageous for increasing variations of signaling properties. We verify the feasibility of this novel heterodimeric artificial receptor platform using cell line-based signaling assays.

## Results

### Construction of a heterodimeric designer c-KIT

**Figure 1:**
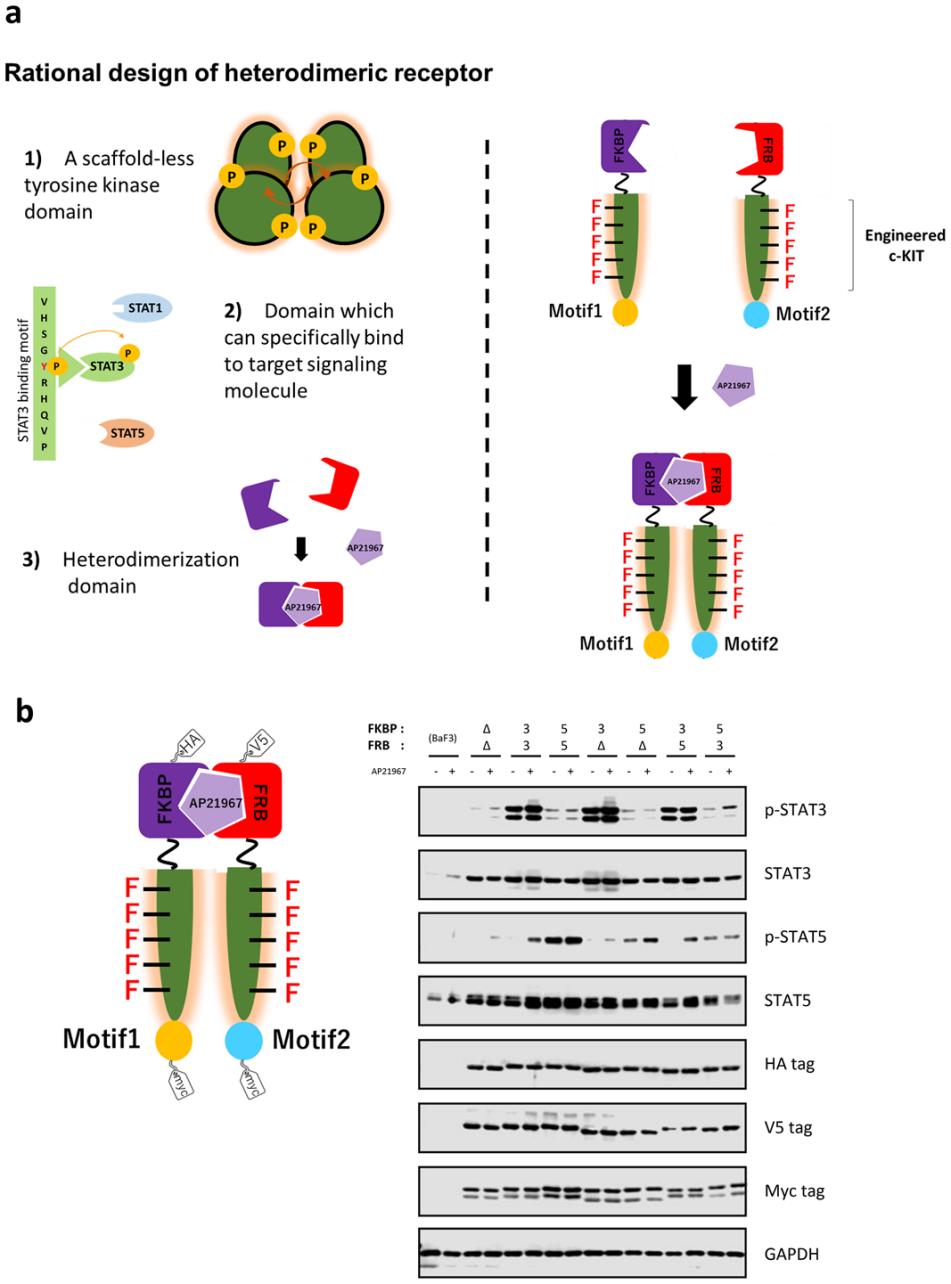
Rational design of heterodimeric designer receptors. (**a**) The designer receptors consist of the following 3 parts; 1) an engineered scaffold-less c-KIT which can phosphorylate the tyrosine motif, 2) a tyrosine motif, which recruits a target signaling molecule when phosphorylated, and 3) a small molecule-responsive heterodimerizing domain (e.g. AP21967-responsive FKBP/FRB_T2098L_ complex). (**b**) The activation profiles of designer receptors without membrane localization. The STAT3- and STAT5-binding motifs were connected on either FKBP chimera or FRB chimera. The symbols Δ, 3, and 5 mean no motif, the STAT3-binding motif, and the STAT5-binding motif, respectively. In western blotting, parental Ba/F3 and the transduced cells were unstimulated (−) or stimulated with 50 nM AP21967 (+). Phospho-STAT3, whole STAT3, phospho-STAT5, whole STAT5, HA tag (FKBP chimera), V5 tag (FRB chimera), Myc tag (both FKBP and FRB chimeras; upper and lower bands, respectively), and GAPDH (loading control) were detected using corresponding primary antibodies. Full-length blots are presented in Supplementary Fig. 8.

We have previously created a designer receptor composed of an engineered c-KIT, a tyrosine motif of interest, and a homodimerization module for activating the c-KIT kinase upon addition of a synthetic small molecule ligand AP20187^23^. The activated engineered kinase domain successfully phosphorylated a tyrosine residue within the tyrosine motif, and consequently the target signaling molecule was recruited to the phosphorylated tyrosine motif. We hypothesized that simply exchanging the homodimerization module of the c-KIT-based designer receptor to a heterodimerization module could create a heterodimeric designer receptor, which would be a versatile platform for designing artificial receptors with a variety of properties. We therefore replaced the homodimeric module (FKBP_F36V_) with wild-type FK506-binding protein 12 (FKBP) or a T2098L mutant of FRB (FRB_T2098L_) in the designer receptor in order to heterodimerically control activation of the receptors with a synthetic small molecule AP21967 (Fig. 1a).

### Non-localized heterodimeric receptors activate two target signaling molecules

As a proof-of-concept experiment, the tyrosine motif was first placed at the C-terminus of the engineered c-KIT, which had been the most versatile position in our previous study demonstrating the homodimeric designer receptors^23^. We selected STAT3 and STAT5 as target signaling molecules to construct heterodimeric designer receptors. IL-3-dependent mouse pro-B Ba/F3 cells were transduced with retroviral vectors encoding either of the heterodimeric designer receptors (either FKBP chimera or FRB_T2098L_ chimera) and either puromycin or blasticidin resistance gene, followed by puromycin and/or blasticidin selection to obtain stable transductants. Cells were stimulated with the FKBP/FRB_T2098L_ heterodimerizing ligand AP21967 and phosphorylation of target signaling molecules STAT3 and STAT5 was detected by Western blotting (Fig. 1b). Consequently, STAT3 and STAT5 (abbreviated as 3 and 5, respectively) were strongly activated in the heterodimeric receptor combinations FK3/FRB3 and FK5/FRB5, in which the same motifs were inserted on both sides of the heterodimeric receptor chains. On the contrary, the target signaling molecules were hardly phosphorylated in the combination FKΔ/FRBΔ, which is a tyrosine motif-null control. Interestingly, phosphorylation of the target signaling molecule was also observed in the combinations FK3/FRBΔ and FK5/FRBΔ, which have the tyrosine motif on only one side of the receptor chains. Furthermore, the target signaling molecules were phosphorylated even when the different tyrosine motifs were placed on both sides of the receptor chains (FK3/FRB5 and FK5/FRB3). However, the phosphorylation levels of STAT3 and most of the phosphorylation levels of STAT5 were ligand-independent, while only p-STAT5 for FK3 /FRB5 was ligand-dependent. This result indicates that the expressed receptors were ligand-independently heterodimerized.

**Figure 2:**
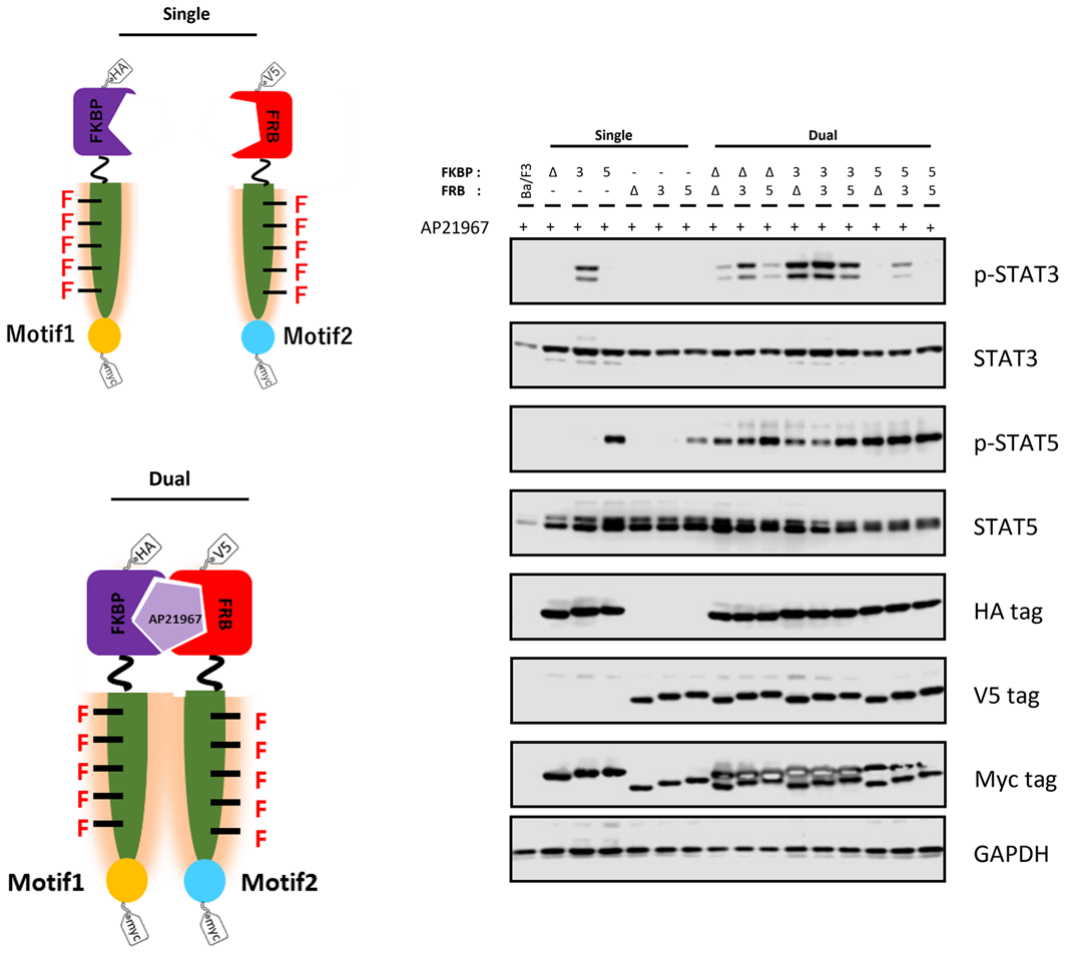
Comparison of the single transductants and double transductants of the FKBP and FRB chimeras. The symbols Δ, 3, and 5 mean no motif, the STAT3-binding motif, and the STAT5-binding motif, respectively. Parental Ba/F3 and the transduced cells were unstimulated (−) or stimulated with 50 nM AP21967 (+). Phospho-STAT3, whole STAT3, phospho-STAT5, whole STAT5, HA tag (FKBP chimera), V5 tag (FRB chimera), Myc tag (both FKBP and FRB chimeras; upper and lower bands, respectively), and GAPDH (loading control) were detected using corresponding primary antibodies. Full-length blots are presented in Supplementary Fig. 8.

We also investigated the phosphorylation of signaling molecules when the FKBP side or FRB side was expressed solely (Fig. 2). Problematically, STAT5 was phosphorylated in a tyrosine motif-independent manner, which may be due to excessive activation of the kinase domain of the heterodimerized designer receptors. Therefore, it is necessary to further modify the designer receptors in order to adjust the kinase activity appropriately.

### Myristoylated heterodimeric receptors diminish the leaky signaling

**Figure 3:**
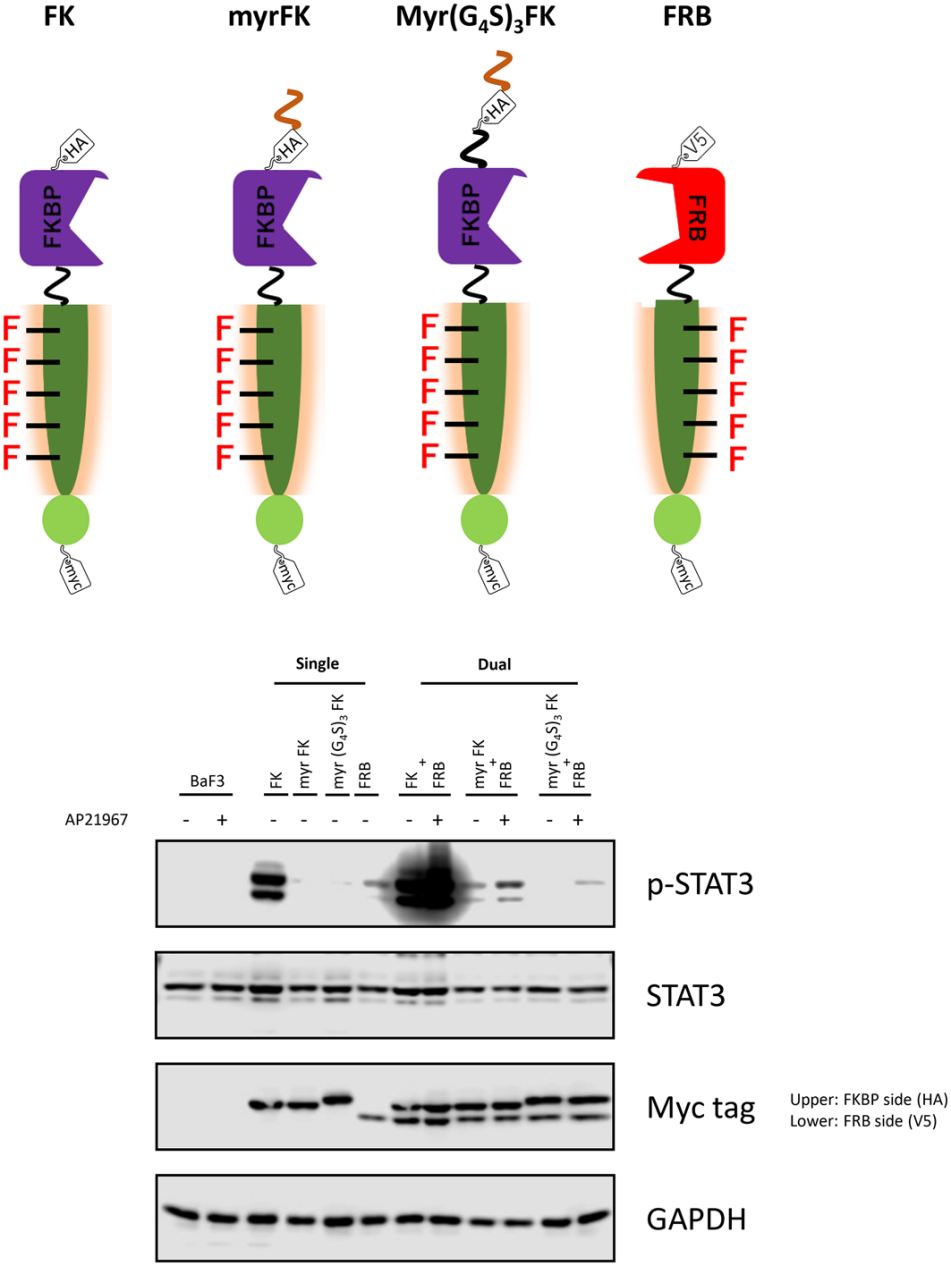
Localization of designer receptors to cell membrane. To mimic natural receptors, the myristoylation signal sequence (myr) with or without the (G_4_S)_3_ flexible linker was appended at the N-terminus of the FKBP (FK) chimera. The single-transduced (Single: FK, myr FK, myr (G_4_S)_3_ FK, FRB), or dual-transduced (Dual: FK + FRB, myr FK + FRB, myr (G_4_S)_3_ FK + FRB) cells were analyzed in western blotting. Parental Ba/F3 and the transduced cells were unstimulated (−) or stimulated with 50 nM AP21967 (+). Phospho-STAT3, whole STAT3, myc tag (both FKBP and FRB chimeras; upper and lower bands, respectively), and GAPDH (loading control) were detected using corresponding primary antibodies. Full-length blots are presented in Supplementary Fig. 8.

In our previous study demonstrating the homodimeric designer receptors, the non-localized receptor exhibited higher expression level and signaling intensity than the membrane-localized receptor, leading to ligand-independent activation. Therefore, to lower the activation levels of the heterodimeric designer receptors, we attached the myristoylation signal sequence with/without a flexible (G_4_S)_3_ linker at the N-terminus of FKBP, and the STAT3-binding motif was linked at both C-termini of the receptors. We investigated whether leakage of signaling could be reduced by this amendment (Fig. 3). As a result, when the myristoylated FKBP chimeras (myr FK and myr (G_4_S)_3_ FK) were expressed solely, the leaky phosphorylation levels of STAT3 were significantly reduced compared to the non-myristoylated FKBP chimera. Furthermore, the myristoylated FKBP chimeras attained weaker signaling intensities and ligand-dependent STAT3 activation when co-expressed with the FRB chimera. Because the myr (G_4_S)_3_ FK chimera was free of signal leakage, this construct was used hereafter for further modifications.

### Motif positions affect the signaling properties of heterodimeric receptors

**Figure 4:**
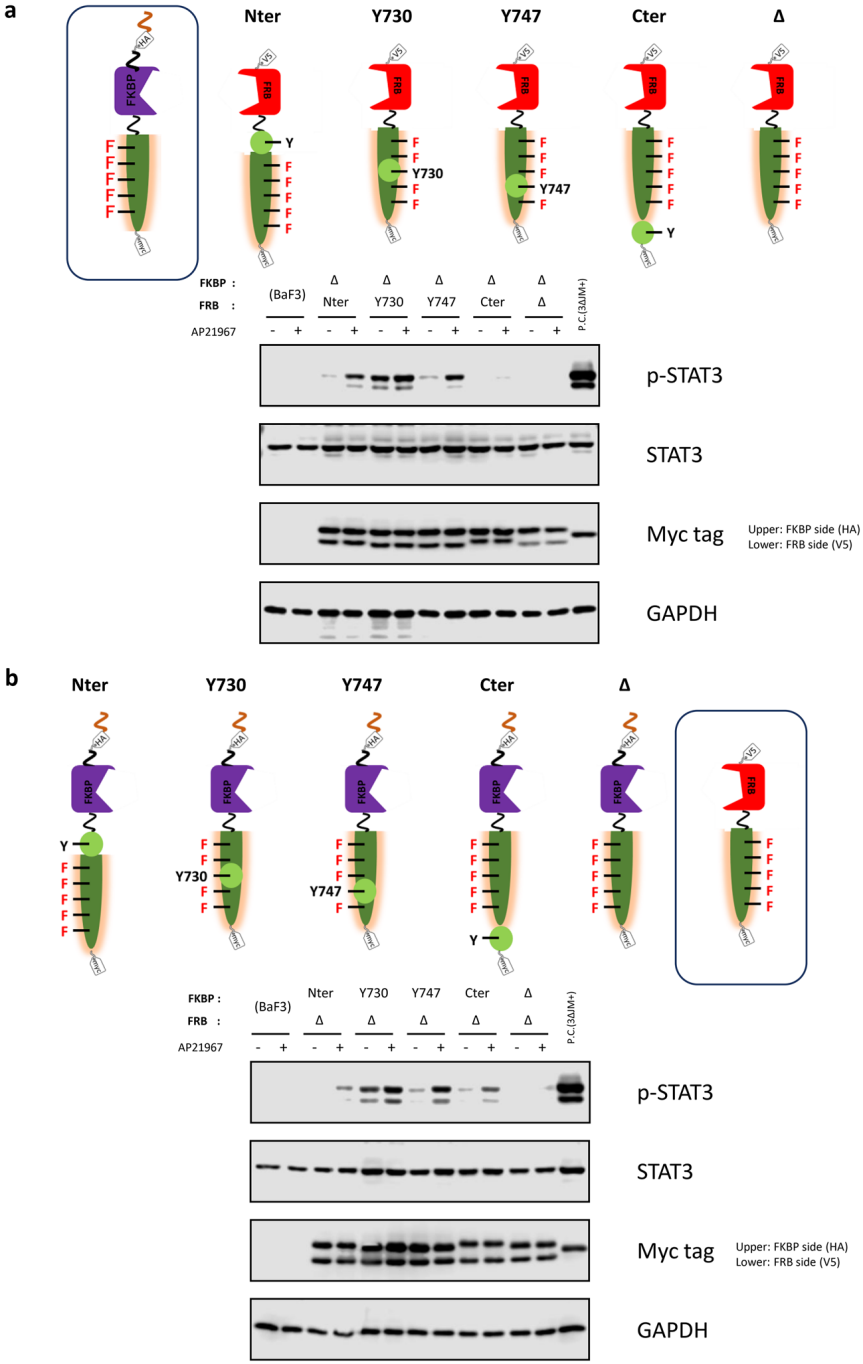
Investigating positional effects for a tyrosine motif. The STAT3-binding motif was positioned at the N-terminus, C-terminus, Y730, or Y747 of the engineered c-KIT of the FRB side (**a**) and FKBP side (**b**) of the chimeras. The symbol Δ means no motif for each position. The positive control (3ΔJM+) is the homodimeric designer receptor, in which the STAT3-binding motif was positioned at the N-terminus of the engineered c-KIT. In western blotting, parental Ba/F3 and the transduced cells were unstimulated (−) or stimulated with 50 nM AP21967 (+). Phospho-STAT3, whole STAT3, myc tag (both FKBP and FRB chimeras; upper and lower bands, respectively), and GAPDH (loading control) were detected using corresponding primary antibodies. Full-length blots are presented in Supplementary Fig. 8.

We hypothesized that the position of the tyrosine motif in the engineered c-KIT might also affect signaling properties. Previously, we demonstrated that tyrosine motifs can be inserted at the N-terminal, Y730, Y747, and C-terminal positions of the engineered c-KIT^23^. Therefore, we investigated the positional effects by inserting the STAT3-binding motif at either of the 4 positions (Fig. 4). When the STAT3-binding motif was attached only to the FRB side, the N-terminal and Y747 positions were superior in terms of ligand dependency and STAT3 phosphorylation levels (Fig. 4a). When the STAT3-binding motif was attached only to the FKBP side, the Y747 and C-terminal positions were superior (Fig. 4b). The results demonstrate that the signaling properties can be adjusted by modulating the position of the tyrosine motif in the heterodimeric designer receptors.

### Construction of optimized heterodimeric receptors

**Figure 5:**
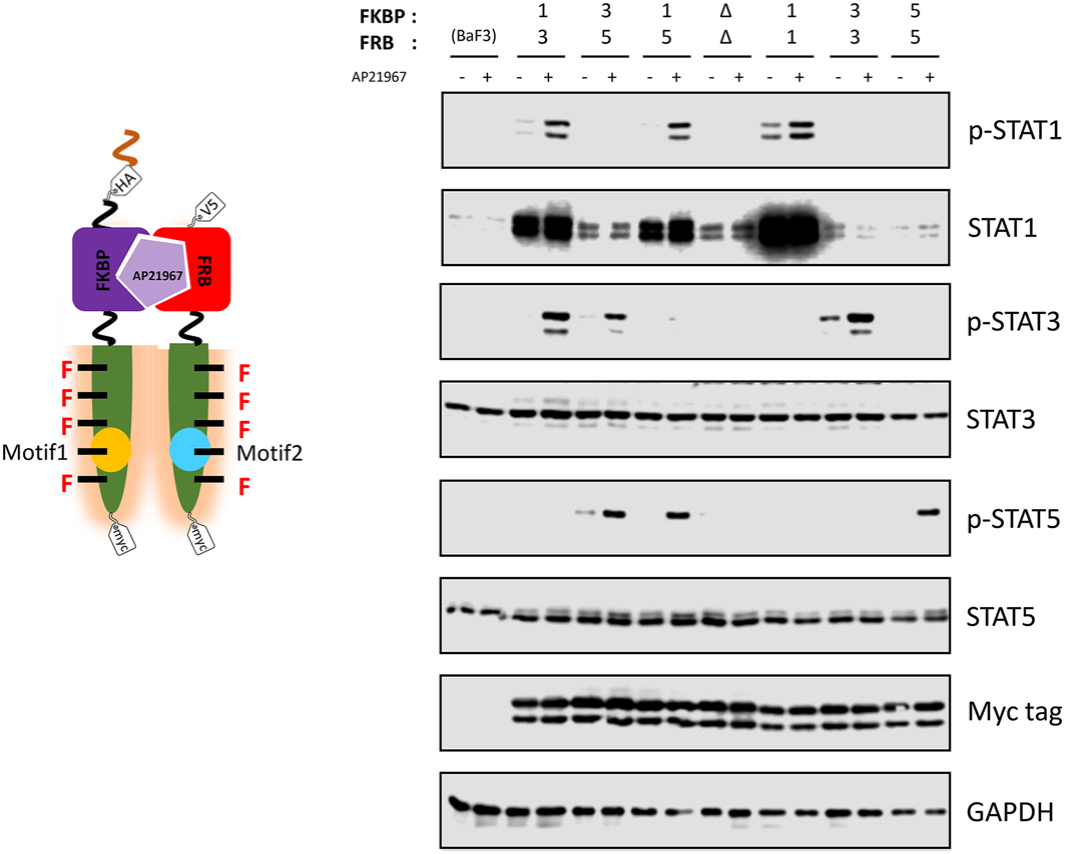
Activation of target signaling molecules using heterodimeric designer receptor. The target signaling molecule-binding motifs were positioned on Y747 of the engineered c-KIT. STAT1-, STAT3-, and STAT5-binding motifs (1, 3, and 5, respectively) were used as the binding motifs. The chimera devoid of tyrosine motifs (Δ) was used as a negative control. In western blotting, parental Ba/F3 and the transduced cells were unstimulated (−) or stimulated with 50 nM AP21967 (+). Phospho-STAT1, whole STAT1, phospho-STAT3, whole STAT3, phospho-STAT5, whole STAT5, myc tag (both FKBP and FRB chimeras; upper and lower bands, respectively), and GAPDH (loading control) were detected using corresponding primary antibodies. Full-length blots are presented in Supplementary Fig. 8.

Because Y747 was the superior position commonly for both FKBP and FRB chimeras, we inserted the STAT1-, STAT3- and STAT5-binding motifs at Y747 in both chimeras and investigated whether the chimeras could activate two target signaling molecules at the same time (Fig. 5). Consequently, when the same tyrosine motifs were placed on both chimeras, only the corresponding signaling molecules were phosphorylated. STAT1 was overexpressed in the transductants with the chimeras incorporating the STAT1-binding motif, which would be due to a positive-feedback regulation of STAT1 as demonstrated in our previous study^20^. Intriguingly, when two different tyrosine motifs were placed separately on the respective heterodimeric chimeras, the two target signaling molecules were phosphorylated without off-target phosphorylation. These results demonstrate that even single tyrosine motif within the heterodimeric receptors is sufficient to activate the target signaling molecule and that the different motifs can be heteromerically embedded for activating multiple signaling molecules in the heterodimeric designer receptors.

### Application to cellular phenotypic assays

**Figure 6:**
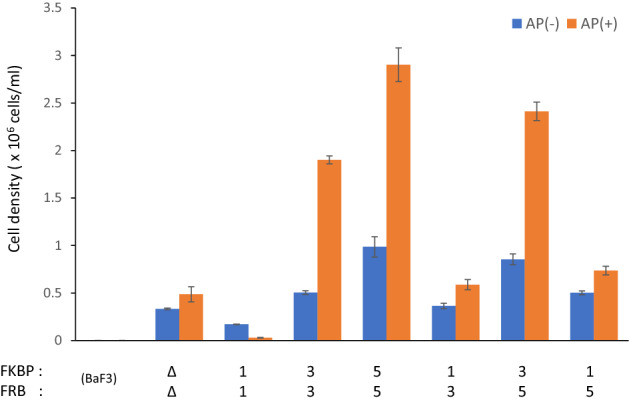
Cell proliferation properties of designer receptor-expressing cells. A cell proliferation assay was performed for the Ba/F3 transductants expressing the designer receptors analyzed in Fig. 5. Cells were seeded into 24-well plates at day 0 (1.0 × 10^5^ cells/ml) and cultured in the medium with/without 50 nM AP21967. On day 3, cell number was counted by flow cytometry. Viable cell densities in triplicate cultures are plotted as mean ± SD.

To examine whether the optimized heterodimeric designer receptors shown in Fig. 5 could be applied to examine cellular phenotype induced by different signaling, a cell proliferation assay was performed. The cells were cultured with or without the heterodimerizing ligand AP21967 for 3 days, and viable cell density was measured by flow cytometry (Fig. 6). As a result, STAT3 and STAT5 promoted cell proliferation, whereas STAT1 suppressed cell proliferation. The heterodimeric receptors simultaneously activating STAT3/STAT5 induced similar levels of cell proliferation to those activating either STAT3 or STAT5. Interestingly, the heterodimeric receptors simultaneously activating STAT3/STAT1 and STAT5/STAT1 induced lower levels of cell proliferation than those activating either STAT3 or STAT5. These results suggest that activation of STAT1 has negative effects on cell proliferation induced by STAT3 or STAT5 in Ba/F3 cells.

## Discussion

In this study, we successfully created heterodimeric designer receptors that can activate target signaling molecules. The heterodimeric designer receptors were constructed by combining three distinct components, namely the heterodimerizing domain, engineered tyrosine kinase (c-KIT) domain, and tyrosine motif. The results proved that the heterodimeric designer receptors can be optimized by rationally assembling the functional components. We also showed that the tyrosine motif-based engineering is a powerful and feasible approach to design artificial receptors with desired signaling properties.

As far as we know, this study is the first report of functional artificial receptors with different tyrosine motifs placed on either side of the receptor chains. Although there are many natural heterodimeric receptors, it has been difficult to mimic them because of their complex dimerization mechanisms. In this study, off-target effects and leaky signaling were problematic when the receptors were expressed in the cytoplasm. When the receptors were anchored to the membrane, the activation levels were weakened but instead the ligand dependency was improved. These results imply that receptors may prefer the vicinity of the membrane for activating on-target signaling molecules without leakage. This notion may partly explain why many natural receptors are localized on the membrane.

Although the FKBP domain does not spontaneously form a homodimer in principle, the results suggest that the FKBP chimeras were dimerized even without ligand stimulation. Furthermore, the propensity of dimerization was also affected by membrane localization; the addition of the myristoylation signal sequence and (G_4_S)_3_ linker drastically diminished ligand-independent dimerization. One possibility is that activation of the kinase domain is restricted by bringing it closer to the membrane, and thus the membrane-localized chimeras become more difficult to be activated than the non-localized ones in the cytoplasm. The position of the tyrosine motif also greatly affected the activation level of the target signaling molecule. The results of Fig. 4 indicated that the motifs at the positions within the kinase insert domain (i.e. Y730 and Y747) were readily activated compared to those at N- and C-termini. This may suggest that strong activation can be obtained in the kinase insert domain even in the natural receptor c-KIT. The superiority or inferiority of the motif position was revealed through this sophisticated receptor construction approach, which highlights the utility of the heterodimeric receptors for elucidating receptor activation mechanisms.

In summary, our study has shown clearly that even heterodimeric artificial receptors can be designed on a motif basis. Subcellular localization and tyrosine motif positions profoundly affected activation profiles of the heterodimeric designer receptors. Finally, we identified a rule for the heterodimeric designer receptors to be functional as intended. This would be a significant advancement in the synthetic engineering of functional receptors and will mark the beginning of the era of tailor-made receptors.

## Materials and methods

### Plasmid construction

The amino acid sequences of the constructed chimeric receptors are provided in Supplementary Information (Supplementary Fig. 1–7). All of the chimeric receptors were subcloned in a retroviral expression plasmid pMK-stuffer-IPTG^24^ or pMK-stuffer-IBTK^24^, which include an internal ribosomal entry site (IRES; I) and puromycin (P) or blasticidin (B) resistance gene cassette, followed by genes encoding self-cleaving T2A peptide (T) and GFP (G) or Kusabira Orange (K). For construction of desired plasmids, pMK-stuffer-IPTG and pMK-stuffer-IBTK were linearized by PCR and the resultant fragments were fused to PCR-amplified insert sequences using In-Fusion HD Cloning Kit (Takara Bio, Shiga, Japan). PrimeSTAR Mutagenesis Basal Kit (Takara Bio) was also used for PCR-based mutagenesis. Transformation of *E. coli* and plasmid extraction were performed in a standard protocol. The plasmids with correct sequences were used for further experiments.

### Cell culture

RPMI 1640 medium (Nissui Pharmaceutical, Tokyo, Japan) supplemented with 10% fetal bovine serum (Sigma-Aldrich, St. Louis, MO), 1 ng/ml murine IL-3 (R&D Systems, Cambridge, MA), 100 U/ml penicillin, and 100 µg/ml streptomycin (Thermo Fisher Scientific, Waltham, MA) was used to culture Ba/F3 cells (RCB0805; RIKEN Cell Bank, Ibaraki, Japan) at 37℃ in a 5% CO_2_ incubator. Dulbecco’s modified Eagle’s medium (DMEM) (Nissui Pharmaceutical) supplemented with 10% fetal bovine serum, 1 µg/ml puromycin (Sigma-Aldrich), and 10 µg/ml blasticidin (Kaken Pharmaceutical, Tokyo, Japan) was used to culture retroviral packaging Plat-E cells at 37℃ in a 10% CO_2_ incubator.

### Retroviral transduction

Retroviral transduction of Ba/F3 cells was conducted similarly to a previous study^25^. Plat-E cells were lipofected with the plasmids using Lipofectamine LTX (Thermo Fisher Scientific) according to the manufacturer’s protocol. After 2 days, Ba/F3 cells were transduced with the viral supernatant pre-adsorbed on RetroNectin (Takara Bio, Shiga, Japan) in 24-well plates. After 2 days, the cells were subjected to selection with 2 µg/ml puromycin and/or 40 µg/ml blasticidin.

### Depletion, stimulation, and lysis of cells

Cells were washed three times with PBS(-) and subsequently cultured in the RPMI medium without IL-3 nor antibiotics for 5 h before ligand stimulation. The cells (1.0 × 10^6^ cells) were harvested by a centrifuge and the cell pellet was resuspended with 500 μl of RPMI medium with or without 50 nM AP21967 (Takara Bio) and incubated at 37℃ for 15 min. The stimulated cells (10^6^ cells) were washed twice with 2 mM Na_3_VO_4_ in ice-cold PBS(-). The cell pellet was lysed with 100 μl lysis buffer (20 mM HEPES (pH 7.5), 150 mM NaCl, 10% glycerol, 1% Triton X-100, 1.5 mM MgCl_2_, 1 mM EGTA, 10 μg/ml aprotinin, 10 μg/ml leupeptin, 1 mM Na_3_VO_4_) and incubated on ice for 10 min. After centrifugation at 21,500 g for 10 min at 4℃, the supernatant was collected, mixed with 33 μl of 4 × Laemmli’s buffer, and boiled at 98℃ for 5 min.

### Signaling analysis

The cell lysates were subjected to SDS-PAGE, then the signaling properties were analyzed by western blotting. The list of rabbit primary antibodies used in this study is described as follows: anti phospho-STAT1 (Y701)(Cell Signaling Technology, Danvers, MA), anti STAT1 (Cell Signaling Technology), anti phospho-STAT3 (Y705) (Cell Signaling Technology), anti STAT3 (Santa Cruz Biotechnology, Santa Cruz, CA), anti phospho-STAT5 (Y694) (Cell Signaling Technology), anti STAT5 (Santa Cruz Biotechnology), anti HA tag (BETHYL), anti V5 tag (Millipore, Burling, MA), anti c-myc tag (BETHYL, Montgomery, TX), and anti GAPDH (Santa Cruz Biotechnology). As a secondary antibody, horseradish peroxidase-conjugated anti rabbit IgG (Thermo Fisher Scientific) was commonly used.

### Cell proliferation assay

To remove IL-3 from the original culture medium, the cells were washed two times by PBS(-) and then seeded into 24-well plates at a density of 1 × 10^5^ cells/ml in the RPMI medium supplemented with/without 50 nM AP20187 under 37℃ in a 5% CO_2_ incubator. Viable cell density was measured after 3 day-incubation in flow cytometry as follows. The cell suspension (100 µl) was transferred to 96-well plates, in which 1 μg/ml propidium iodide, 4 µl Flow-Count beads (Beckman Coulter, Brea, CA), and 46 µl PBS were added to each well in advance. The number of cells was measured using a FACSCalibur flow cytometer (Becton–Dickinson, Franklin Lakes, NJ). Viable cells were identified as propidium iodide-negative cells using FlowJo software (Becton–Dickinson).

## Supplementary Information

Supplementary Information.
