# Supplementary material for: Rational design of heterodimeric receptors capable of activating target signaling molecules
Source: Sci Rep. 2021 Aug 19;11:16809. doi: 10.1038/s41598-021-96396-3 (PMC8376883; doi:10.1038/s41598-021-96396-3)
Supplement: Supplementary file 1 — Supplementary Information. [file 41598_2021_96396_MOESM1_ESM.pdf]

Supplementary Information

Rational design of heterodimeric receptors capable of activating target signaling molecules

Tatphon Kongkrongtong[1], Ruolan Zhang[2], Masahiro Kawahara[1,2,3,*]

Table of Contents

1. **Supplementary Figure 1** S2
2. **Supplementary Figure 2** S3
3. **Supplementary Figure 3** S4
4. **Supplementary Figure 4** S5
5. **Supplementary Figure 5** S6
6. **Supplementary Figure 6** S7
7. **Supplementary Figure 7** S8-S9
8. **Supplementary Figure 8** S10-S14

STAT1 binding motif: PTSFGYDKPHVL

STAT3 binding motif: VVHSGYRHQVPS

STAT5 binding motif: LMDNAYFCEAD

Supplementary Figure 1. Amino acid sequence of binding motifs.

FKBP side: pMK-HA-FKBP-$(G_4S)_3$-engineered c-KIT-binding motif-myc-IRES-Puro^R-T2A-EGFP

HA tag
FKBP
$(G_4S)_3$ linker
Engineered c-KIT
binding motif 1
myc tag

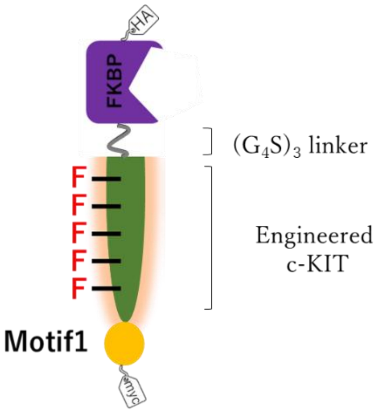

MYPYDVPDYAGSGGVQVETISPGDGRTFPKRGQTCVVHYTGMLEDGKKFDSSRDRNKPF

KFMLGKQEVIRGWEEGVAQMSVGQRAKLTISPDYAYGATGHPGIIPPHATLVFDVELLKLE

GSGGGGGSGGGGSGGGGSGRVDPTQLPYDHKWEFPRNRLSFGKTLGAGAFGKVVEATAY

GLIKSDAAMTVAVKMLKPSAHLTEREALMSELKVLSYLGNHMNIVNLLGACTIGGPTLVITE

YCCYGDLLNFLRRKRDSFICSKQEDHAEAALFKNLLHSKESSCSDSTNEFMDMKPGVSFVVP

TKADKRRSVRIGSFIERDVTPAIMEDDELALDLEDLLSFSYQVAKGMAFLASKNCIHRDLAA

RNILLTHGRITKICDFGLARDIKNDSNYVVKGNARLPVKWMAPESIFNCVYTFESDVWSYGI

FLWELFSLGSSPYPGMPVDSKFYKMIKEGFRMLSPEHAPAEMYDIMKTCWDADPLKRPTFK

QIVQLIEKQISESTNHIFSNLANCSPNRQKPVVDHSVRINSVGSTASSSQPLLVHDDVbinding

_motif_1IDEQKLISEEDL*

Supplementary Figure 2. Amino acid sequence of heterodimeric designer receptors (FKBP side) tested in main Figure 1. Black; HA tag or myc tag, Purple; FKBP, Gray; $(GGGGS)_3$ linker or other linker sequences, Dark green; Engineered c-KIT, Yellow; binding motif 1, Red; tyrosine-to-phenylalanine mutation

FRB side: pMK-V5-FRB$_{T2098L}$-$(G_4S)_3$-engineered c-KIT-binding motif-myc-IRES-Blasticidin^R-T2A-KO

- V5 tag
- FRB$_{T2098L}$
- $(G_4S)_3$ linker
- Engineered c-KIT
- binding motif 2
- myc tag

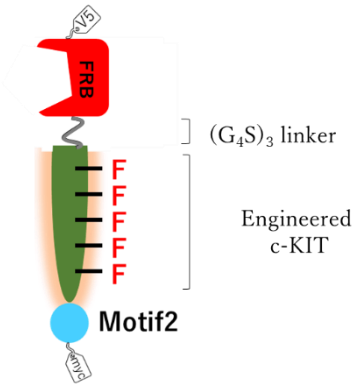

MGKPIPNPLLGLDSTGSGILWHEMWHEGLEEASRLYFGERNVKGMFEVLEPLHAMMERGP

QTLKETSFNQAYGRDLMEAQEWCRKYMKSGNVKDLLQAWDLYYHVFRRISKGSGGGGGS

GGGGSGGGGSGRVDPTQLPYDHKWEFPRNRLSFGKTLGAGAFGKVVEATAYGLIKSDAA

MTVAVKMLKPSAHLTEREALMSELKVLSYLGNHMNIVNLLGACTIGGPTLVITEYCCYGDL

LNFLRRKRDSFICSKQEDHAEAALFKNLLHSKESSCSDSTNEFMDMKPGVSFVVPTKADKRR

SVRIGSFIERDVTPAIMEDDELALDLEDLLSFSYQVAKGMAFLASKNCIHRDLAARNILLTHG

RITKICDFGLARDIKNDSNYVVKGNARLPVKWMAPESIFNCVYTFESDVWSYGIFLWELFSL

GSSPYPGMPVDSKFYKMIKEGFRMLSPEHAPAEMYDIMKTCWDADPLKRPTFKQIVQLIEK

QISESTNHIFSNLANCSPNRQKPVVDHSVRINSVGSTASSSQPLLVHDDVbinding_motif_2ID

EQKLISEEDL*

Supplementary Figure 3. Amino acid sequence of heterodimeric designer receptors (FRB side) tested in main Figure 1. Black; V5 tag or myc tag, Vermillion; FRB$_{T2098L}$, Gray; $(GGGGS)_3$ linker or other linker sequences, Dark green; Engineered c-KIT, Light blue; binding motif 2, Red; tyrosine-to-phenylalanine mutation

FKBP side: pMK-myr-HA-FKBP-$(G_4S)_3$-engineered c-KIT-binding motif-myc-IRES-Puro^R-T2A-EGFP

- Myristoylation signal
- HA tag
- FKBP
- $(G_4S)_3$ linker
- Engineered c-KIT
- STAT3 binding motif
- myc tag

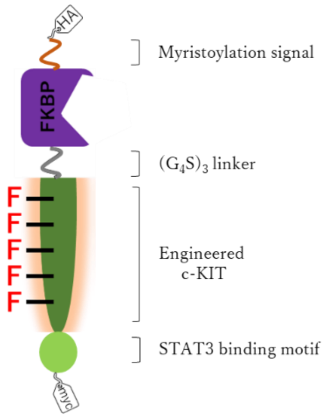

MGSSKSKPKDPSQRYPYDVPDYAGSGGVQVETISPGDGRTFPKRGQTCVVHYTGMLEDGK
KFDSSRDRNKPFKFMLGKQEVIRGWEEGVAQMSVGQRAKLTISPDYAYGATGHPGIIPPHA
TLVFDVELLKLEGSGGGGGSGGGGSGGGGSGRVDPTQLPYDHKWEFPRNRLSFGKTLGA
GAFGKVVEATAYGLIKSDAAMTVAVKMLKPSAHLTEREALMSELKVLSYLGNHMNIVNLLG
ACTIGGPTLVITEYCCYGDLLNFLRRKRDSFICSKQEDHAEAALFKNLLHSKESSCSDSTNEF
MDMKPGVSFVVPTKADKRRSVRIGSFIERDVTPAIMEDDELALDLEDLLSFSYQVAKGMAFL
ASKNCIHRDLAARNILLTHGRITKICDFGLARDIKNDSNYVVKGNARLPVKWMAPESIFNCV
YTFESDVWSYGIFLWELFSLGSSPYPGMPVDSKFYKMIKEGFRMLSPEHAPAEMYDIMKTC
WDADPLKRPTFKQIVQLIEKQISESTNHIFSNLANCSPNRQKPVVDHSVRINSVGSTASSSQP
LLVHDDVVVHSGYRHQVPSIDEQKLISEEDL*

Supplementary Figure 4. Amino acid sequence of heterodimeric designer receptors (myr FK) tested in main Figure 2. Magenta; Myristoylation signal, Black; HA tag or myc tag, Purple; FKBP, Gray; $(GGGGS)_3$ linker or other linker sequences, Dark green; Engineered c-KIT, Light green; STAT3-binding motif, Red; tyrosine-to-phenylalanine mutation

FKBP side: pMK-myr-HA-$(G_4S)_3$-FKBP-$(G_4S)_3$-engineered c-KIT-binding motif-myc-IRES-Puro^R-T2A-EGFP

- Myristoylation signal
- HA tag
- $(G_4S)_3$ linker
- FKBP
- $(G_4S)_3$ linker
- Engineered c-KIT
- STAT3 binding motif
- myc tag

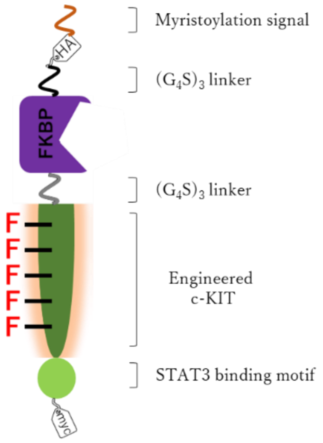

MGSSKSKPKDPSQRYPYDVPDYAGSGGGGGSGGGGSGGGGSGSGGVQVETISPGDGRTFP

KRGQTCVVHYTGMLEDGKKFDSSRDRNKPFKFMLGKQEVIRGWEEGVAQMSVGQRAKLT

ISPDYAYGATGHPGIIPPHATLVFDVELLKLEGSGGGGGSGGGGSGGGGSGRVDPTQLPYD

HKWEFPRNRLSFGKTLGAGAFGKVVEATAYGLIKSDAAMTVAVKMLKPSAHLTEREALMSE

LKVLSYLGNHMNIVNLLGACTIGGPTLVITEYCCYGDLLNFLRRKRDSFICSKQEDHAEAAL

FKNLLHSKESSCSDSTNEFMDMKPGVSFVVPTKADKRRSVRIGSFIERDVTPAIMEDDELAL

DLEDLLSFSYQVAKGMAFLASKNCIHRDLAARNILLTHGRITKICDFGLARDIKNDSNYVVK

GNARLPVKWMAPESIFNCVYTFESDVWSYGIFLWELFSLGSSPYPGMPVDSKFYKMIKEGFR

MLSPEHAPAEMYDIMKTCWDADPLKRPTFKQIVQLIEKQISESTNHIFSNLANCSPNRQKPV

VDHSVRINSVGSTASSSQPLLVHDDVVVHSGYRHQVPSIDEQKLISEEDL*

Supplementary Figure 5. Amino acid sequence of heterodimeric designer receptors (myr $(G_4S)_3$ FK) tested in main Figure 2. Magenta; Myristoylation signal, Black; HA tag or myc tag, Purple; FKBP, Gray; $(GGGGS)_3$ linker or other linker sequences, Dark green; Engineered c-KIT, Light green; STAT3-binding motif, Red; tyrosine-to-phenylalanine mutation

FRB side: pMK-V5-FRB_{T2098L}-$(G_4S)_3$-engineered c-KIT-binding motif-myc-IRES-$Blasticidin^R$-T2A-KO

- V5 tag
- FRB_{T2098L}
- $(G_4S)_3$ linker
- Engineered c-KIT
- STAT3 binding motif
- myc tag

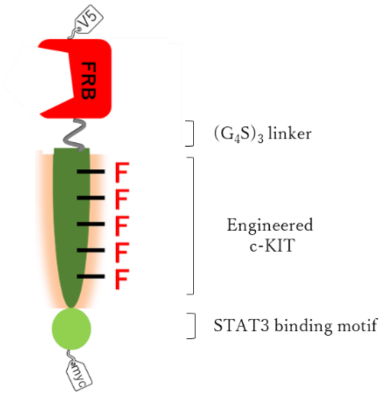

MGKPIPNPLLGLDSTGSGILWHEMWHEGLEEASRLYFGERNVKGMFEVLEPLHAMMERGP
QTLKETSFNQAYGRDLMEAQEWCRKYMKSGNVKDLLQAWDLYYHVFRRISKGSGGGGGS
GGGGSGGGGSGRVDPTQLPYDHKWEFPRNRLSFGKTLGAGAFGKVVEATAYGLIKSDAA
MTVAVKMLKPSAHLTEREALMSELKVLSYLGNHMNIVNLLGACTIGGPTLVITEYCCYGDL
LNFLRRKRDSFICSKQEDHAEAALFKNLLHSKESSCSDSTNEFMDMKPGVSFVVPTKADKRR
SVRIGSFIERDVTPAIMEDDELALDLEDLLSFSYQVAKGMAFLASKNCIHRDLAARNILLTHG
RITKICDFGLARDIKNDSNYVVKGNARLPVKWMAPESIFNCVYTFESDVWSYGIFLWELFSL
GSSPYPGMPVDSKFYKMIKEGFRMLSPEHAPAEMYDIMKTCWDADPLKRPTFKQIVQLIEK
QISESTNHIFSNLANCSPNRQKPVVDHSVRINSVGSTASSSQPLLVHDDVVVHSGYRHQVPSI
DEQKLISEEDL*

Supplementary Figure 6. Amino acid sequence of heterodimeric designer receptors (FRB side) tested in main Figure 2. Black; V5 tag or myc tag, Vermillion; FRB_{T2098L}, Gray; $(GGGGS)_3$ linker or other linker sequences, Dark green; Engineered c-KIT, Light green; STAT3-binding motif, Red; tyrosine-to-phenylalanine mutation

binding motif

Engineered c-KIT

Binding motif at the N-terminus of engineered-c-KIT (Nter)

binding_motifDPTQLPYDHKWEFPRNRLSFGKTLGAGAFGKVVEATAYGLIKSDAAMTVAV
KMLKPSAHLTEREALMSELKVLSYLGNHMNIVNLLGACTIGGPTLVITEYCCYGDLLNFLRR
KRDSFICSKQEDHAEAALFKNLLHSKESSCSDSTNEFMDMKPGVSFVVPTKADKRRSVRIGS
FIERDVTPAIMEDDELALDLEDLLSFSYQVAKGMAFLASKNCIHRDLAARNILLTHGRITKIC
DFGLARDIKNDSNYVVKGNARLPVKWMAPESIFNCVYTFESDVWSYGIFLWELFSLGSSPYP
GMPVDSKFYKMIKEGFRMLSPEHAPAEMYDIMKTCWDADPLKRPTFKQIVQLIEKQISEST
NHIFSNLANCSPNRQKPVVDHSVRINSVGSTASSSQPLLVHDDV

Binding motif at Y730 of engineered-c-KIT (Y730)

DPTQLPYDHKWEFPRNRLSFGKTLGAGAFGKVVEATAYGLIKSDAAMTVAVKMLKPSAHL
TEREALMSELKVLSYLGNHMNIVNLLGACTIGGPTLVITEYCCYGDLLNFLRRKRDSFICSK
QEDHAEAALFKNLLHSKESSCSDSTNEFMDMbinding_motifDKRRSVRIGSFIERDVTPAIME
DDELALDLEDLLSFSYQVAKGMAFLASKNCIHRDLAARNILLTHGRITKICDFGLARDIKND
SNYVVKGNARLPVKWMAPESIFNCVYTFESDVWSYGIFLWELFSLGSSPYPGMPVDSKFYKM
IKEGFRMLSPEHAPAEMYDIMKTCWDADPLKRPTFKQIVQLIEKQISESTNHIFSNLANCSPN
RQKPVVDHSVRINSVGSTASSSQPLLVHDDV

Binding motif at Y747 of engineered-c-KIT (Y747)

DPTQLPYDHKWEFPRNRLSFGKTLGAGAFGKVVEATAYGLIKSDAAMTVAVKMLKPSAHL
TEREALMSELKVLSYLGNHMNIVNLLGACTIGGPTLVITEYCCYGDLLNFLRRKRDSFICSK
QEDHAEAALFKNLLHSKESSCSDSTNEFMDMKPGVSFVVPTKADKRRSbinding_motifPAIM
EDDELALDLEDLLSFSYQVAKGMAFLASKNCIHRDLAARNILLTHGRITKICDFGLARDIKN

DSNYVVKGNARLPVKWMAPESIFNCVYTFESDVWSYGIFLWELFSLGSSPYPGMPVDSKFYK

MIKEGFRMLSPEHAPAEMYDIMKTCWDADPLKRPTFKQIVQLIEKQISESTNHIFSNLANCS

PNRQKPVVDHSVRINSVGSTASSSQPLLVHDDV

Binding motif at the C-terminus of engineered-c-KIT (Cter)

DPTQLPYDHKWEFPRNRLSFGKTLGAGAFGKVVEATAYGLIKSDAAMTVAVKMLKPSAHL

TEREALMSELKVLSYLGNHMNIVNLLGACTIGGPTLVITEYCCYGDLLNFLRRKRDSFICSK

QEDHAEAALFKNLLHSKESSCSDSTNEFMDMKPGVSFVVPTKADKRRSVRIGSFIERDVTPA

IMEDDELALDLEDLLSFSYQVAKGMAFLASKNCIHRDLAARNILLTHGRITKICDFGLARDIK

NDSNYVVKGNARLPVKWMAPESIFNCVYTFESDVWSYGIFLWELFSLGSSPYPGMPVDSKFY

KMIKEGFRMLSPEHAPAEMYDIMKTCWDADPLKRPTFKQIVQLIEKQISESTNHIFSNLANC

SPNRQKPVVDHSVRINSVGSTASSSQPLLVHDDVbinding_motif

Engineered-c-KIT (No motif; Δ)

DPTQLPYDHKWEFPRNRLSFGKTLGAGAFGKVVEATAYGLIKSDAAMTVAVKMLKPSAHL

TEREALMSELKVLSYLGNHMNIVNLLGACTIGGPTLVITEYCCYGDLLNFLRRKRDSFICSK

QEDHAEAALFKNLLHSKESSCSDSTNEFMDMKPGVSFVVPTKADKRRSVRIGSFIERDVTPA

IMEDDELALDLEDLLSFSYQVAKGMAFLASKNCIHRDLAARNILLTHGRITKICDFGLARDIK

NDSNYVVKGNARLPVKWMAPESIFNCVYTFESDVWSYGIFLWELFSLGSSPYPGMPVDSKFY

KMIKEGFRMLSPEHAPAEMYDIMKTCWDADPLKRPTFKQIVQLIEKQISESTNHIFSNLANC

SPNRQKPVVDHSVRINSVGSTASSSQPLLVHDDV

Supplementary Figure 7. Amino acid sequence of engineered c-KIT variants with differential positions of the binding motif tested in main Figures 3 and 4. Yellow; Binding motif, Dark Green; Engineered c-KIT, Red; tyrosine-to-phenylalanine mutation

Supplementary Figure 8. Uncropped blot images.

FIG. 1

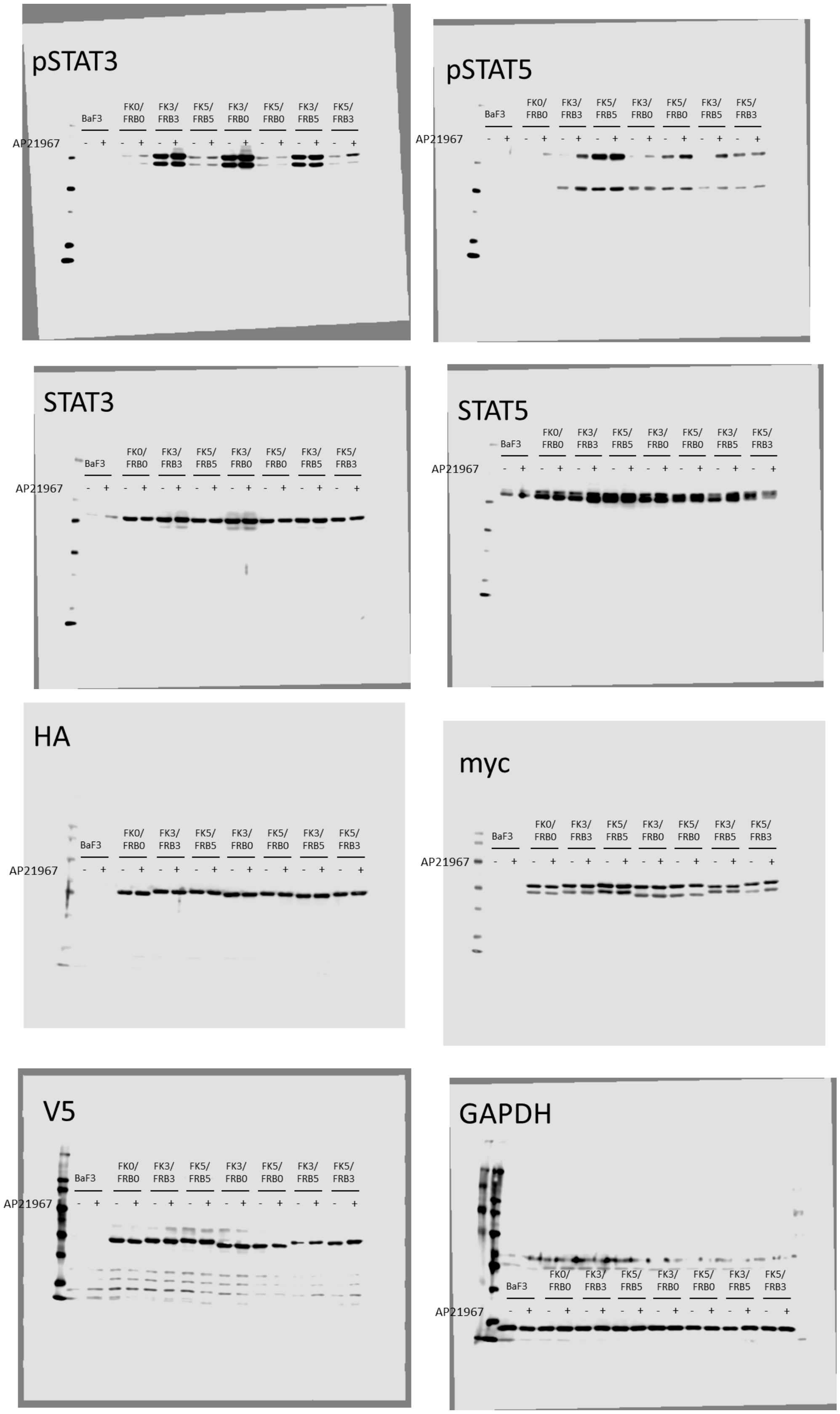

FIG. 2

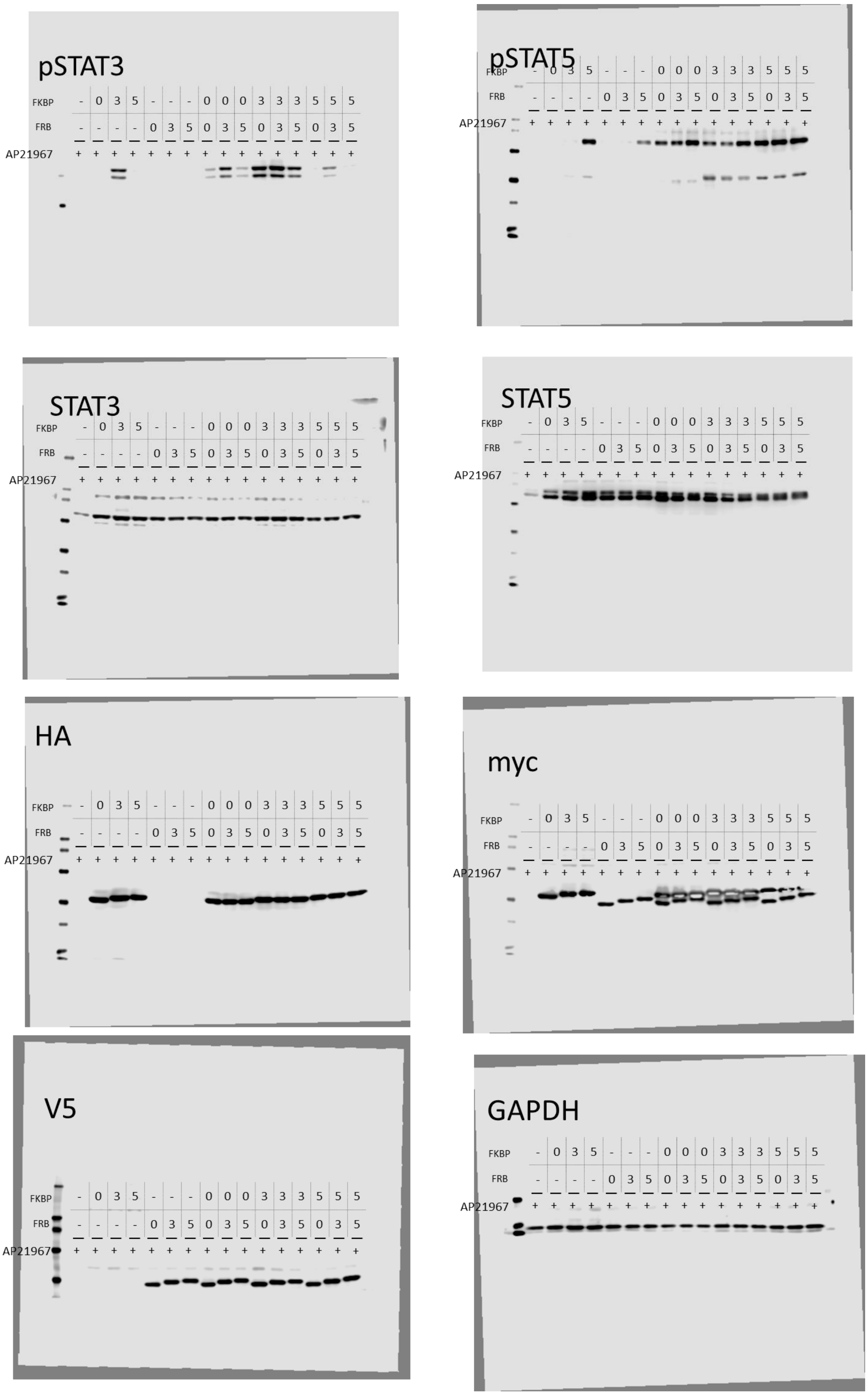
pSTAT3
FKBP - 0 3 5 - - - 0 0 0 3 3 3 5 5 5
FRB - - - - 0 3 5 0 3 5 0 3 5 0 3 5
AP21967 + + + + + + + + + + + + + + + +
pSTAT5
FKBP - 0 3 5 - - - 0 0 0 3 3 3 5 5 5
FRB - - - - 0 3 5 0 3 5 0 3 5 0 3 5
AP21967 + + + + + + + + + + + + + + + +
STAT3
FKBP - 0 3 5 - - - 0 0 0 3 3 3 5 5 5
FRB - - - - 0 3 5 0 3 5 0 3 5 0 3 5
AP21967 + + + + + + + + + + + + + + + +
STAT5
FKBP - 0 3 5 - - - 0 0 0 3 3 3 5 5 5
FRB - - - - 0 3 5 0 3 5 0 3 5 0 3 5
AP21967 + + + + + + + + + + + + + + + +
HA
FKBP - 0 3 5 - - - 0 0 0 3 3 3 5 5 5
FRB - - - - 0 3 5 0 3 5 0 3 5 0 3 5
AP21967 + + + + + + + + + + + + + + + +
myc
FKBP - 0 3 5 - - - 0 0 0 3 3 3 5 5 5
FRB - - - - 0 3 5 0 3 5 0 3 5 0 3 5
AP21967 + + + + + + + + + + + + + + + +
V5
FKBP - 0 3 5 - - - 0 0 0 3 3 3 5 5 5
FRB - - - - 0 3 5 0 3 5 0 3 5 0 3 5
AP21967 + + + + + + + + + + + + + + + +
GAPDH
FKBP - 0 3 5 - - - 0 0 0 3 3 3 5 5 5
FRB - - - - 0 3 5 0 3 5 0 3 5 0 3 5
AP21967 + + + + + + + + + + + + + + + +

FIG. 3

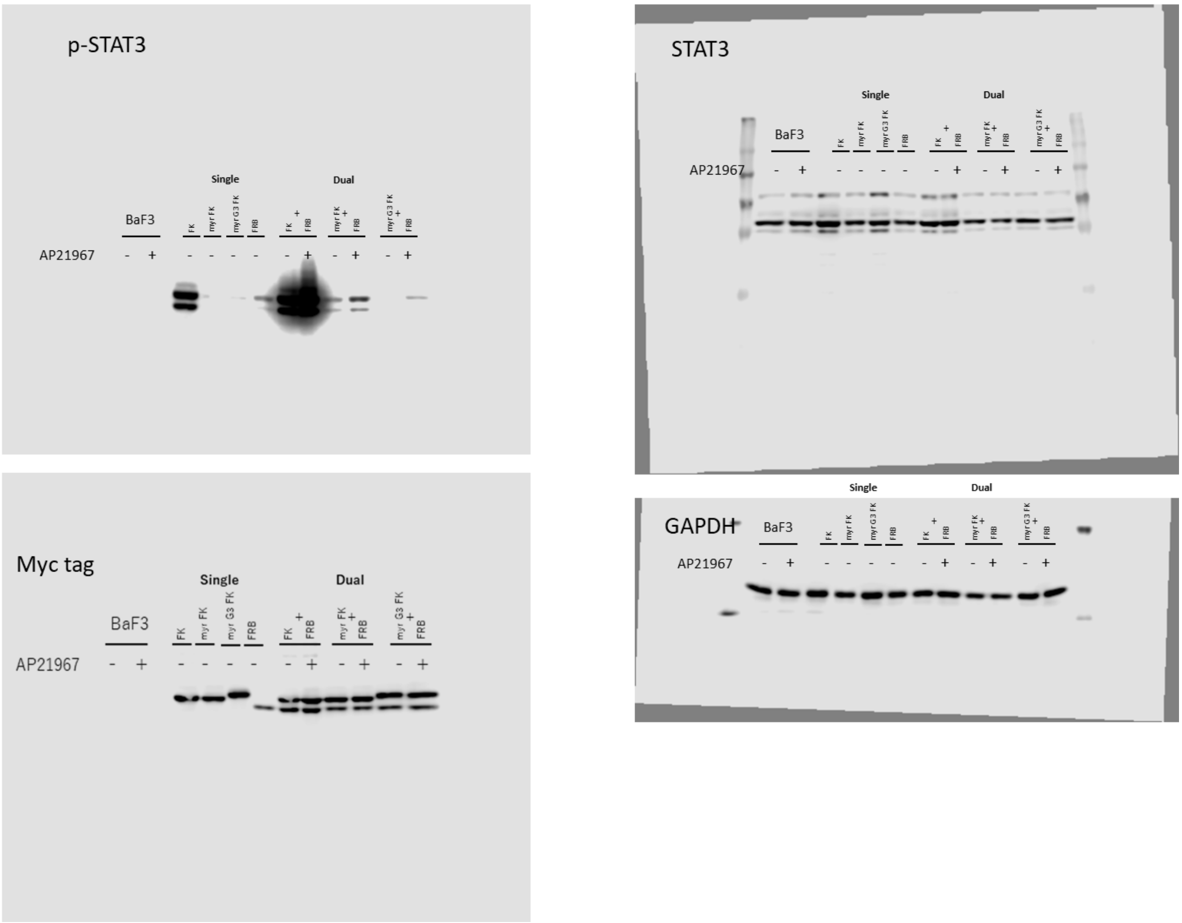

FIG. 4

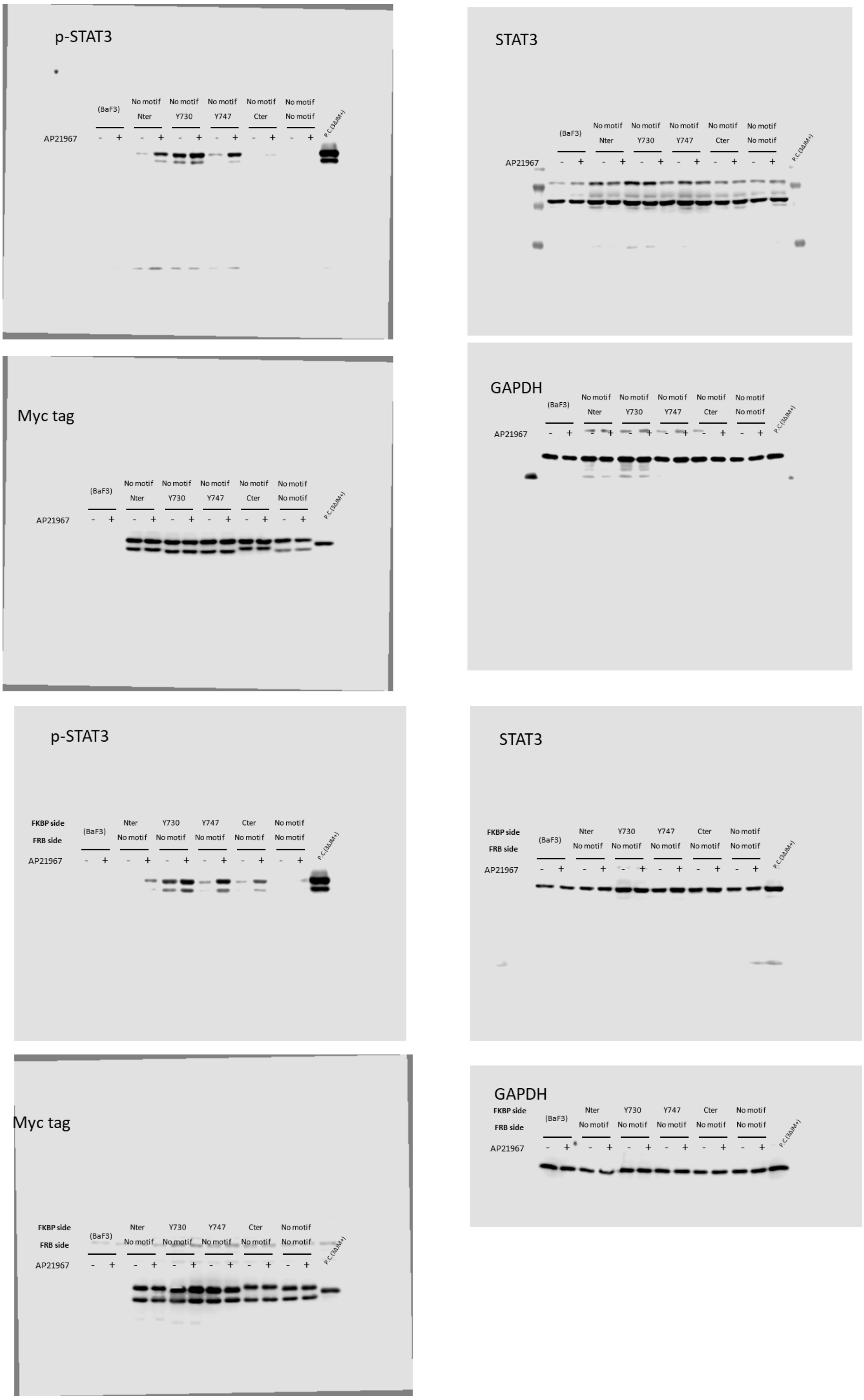
p-STAT3
STAT3
Myc tag
GAPDH
(BaF3)
No motif
Nter
Y730
Y747
Cter
FKBP side
FRB side
AP21967
- + - + - + - + - + - +

FIG. 5

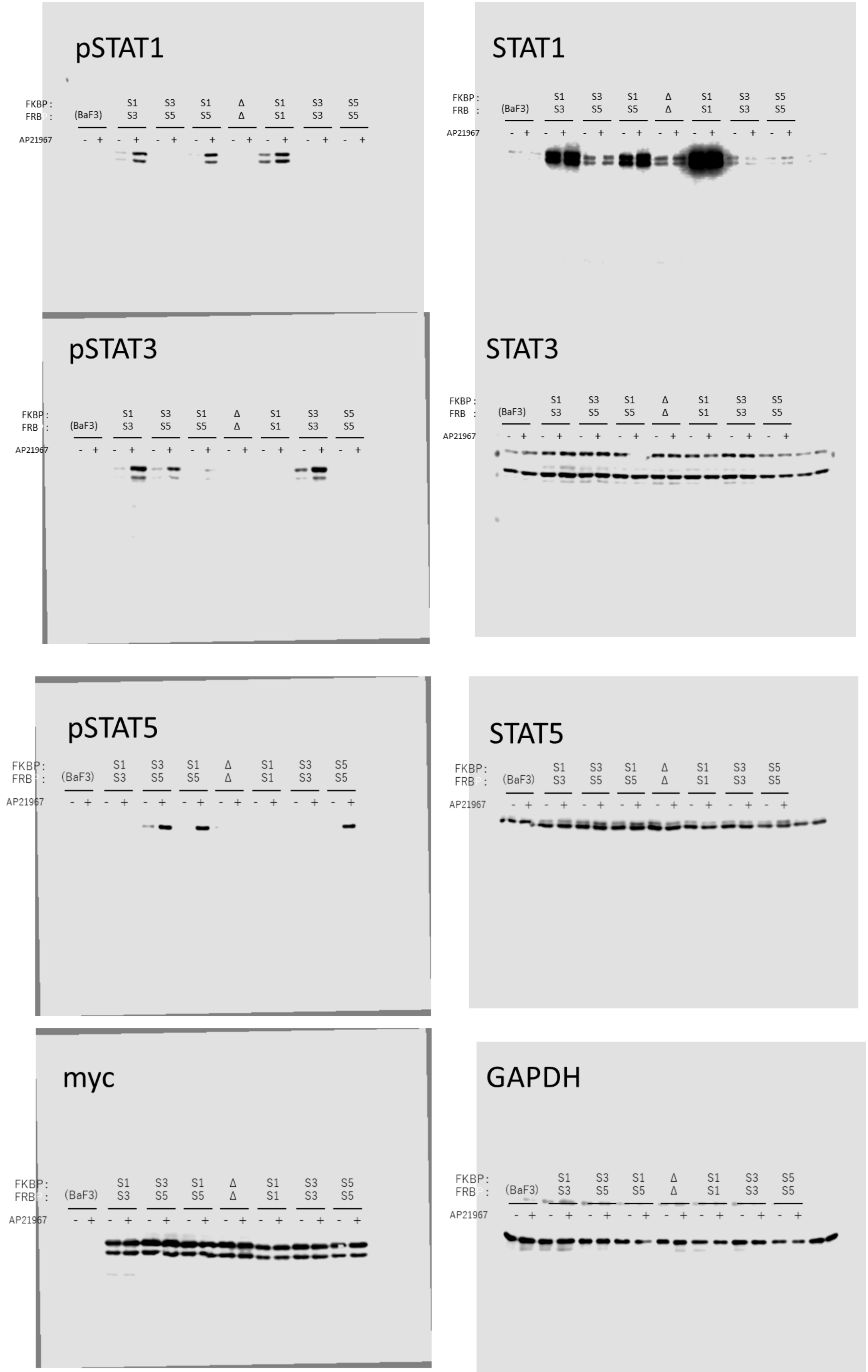
pSTAT1
STAT1
pSTAT3
STAT3
pSTAT5
STAT5
myc
GAPDH
FKBP: (BaF3) S1 S3 S1 Δ S1 S3 S5
FRB: S3 S5 S5 Δ S1 S3 S5
AP21967 - + - + - + - + - + - + - + - +
